# Medicinal potential of mycelium and fruiting bodies of an arboreal mushroom *Fomitopsis officinalis* in therapy of lifestyle diseases

**DOI:** 10.1038/s41598-020-76899-1

**Published:** 2020-11-18

**Authors:** Agata Fijałkowska, Bożena Muszyńska, Katarzyna Sułkowska-Ziaja, Katarzyna Kała, Anna Pawlik, Dawid Stefaniuk, Anna Matuszewska, Kamil Piska, Elżbieta Pękala, Piotr Kaczmarczyk, Jacek Piętka, Magdalena Jaszek

**Affiliations:** 1 Jagiellonian University Medical College, Faculty of Pharmacy, Department of Pharmaceutical Botany, 9 Medyczna Street, 30-688 Kraków, Poland; 2 Chair of Biochemistry and Biotechnology, Faculty of Biology and Biotechnology, Institute of Biological Sciences, Maria Curie-Skłodowska University, 19 Akademicka Street, 20-033 Lublin, Poland; 3 Jagiellonian University Medical College, Faculty of Pharmacy, Department of Pharmaceutical Biochemistry, 9 Medyczna Street, 30-688 Kraków, Poland; 4 Higher School of Medicine in Sosnowiec, 6 Wojska Polskiego Street, 41-200 Sosnowiec, Poland; 5 Department of Forest Protection, Institute of Forest Sciences, Warsaw University of Life Sciences – SGGW, 159 Nowoursynowska Street, 02-776 Warsaw, Poland

**Keywords:** Biochemistry, Biological techniques, Biotechnology, Cancer, Cell biology, Drug discovery, Immunology, Molecular biology, Physiology, Biomarkers, Diseases, Health care, Medical research, Oncology

## Abstract

*Fomitopsis officinalis* is a medicinal mushroom used in traditional European eighteenth and nineteenth century folk medicine. Fruiting bodies of *F. officinalis* were collected from the natural environment of Świętokrzyskie Province with the consent of the General Director for Environmental Protection in Warsaw. Mycelial cultures were obtained from fragments of *F. officinalis* fruiting bodies. The taxonomic position of the mushroom mycelium was confirmed using the PCR method. The presence of organic compounds was determined by HPLC–DAD analysis. Bioelements were determined by AF-AAS. The biochemical composition of the tested mushroom material was confirmed with the FTIR method. Antioxidant properties were determined using the DPPH method, and the antiproliferative activity was assessed with the use of the MTT test. The presence of indole compounds (l-tryptophan, 6-methyl-d,l-tryptophan, melatonin, 5-hydroxy-l-tryptophan), phenolic compounds (*p*-hydroxybenzoic acid, gallic acid, catechin, phenylalanine), and sterols (ergosterol, ergosterol peroxide) as well as trace elements was confirmed in the mycelium and fruiting bodies of *F. officinalis*. Importantly, a high level of 5-hydroxy-l-tryptophan in in vitro mycelium cultures (517.99 mg/100 g d.w) was recorded for the first time. The tested mushroom extracts also showed antioxidant and antiproliferative effects on the A549 lung cancer cell line, the DU145 prostate cancer cell line, and the A375 melanoma cell line.

## Introduction

*Fomitopsis officinalis* (Vill.) Bondartsev & Singer (quinine conk) is an arboreal mushroom species belonging to the Fomitopsidaceae family and Polyporales order. Given the rarity of its occurrence, it has been under strict species protection since 1983. It was also placed on “The IUCN Red List of Threatened Species” as a species at risk of extinction and included in the list of 33 endangered species in Europe^1^.

The species is a parasite living on dead and alive coniferous tree trunks and causes intensive brown rot of wood. It can be found mostly on trees belonging to the *Larix* spp. and more seldom on trees of *Abies* spp., *Pinus* spp., *Picea* spp., *Cydrus* spp., *Tsuga* spp., and *Pseudotsuga* spp.^2^.

Due to its medicinal effects, *F. officinalis* was increasingly gaining popularity among eighteenth and nineteenth-century pharmacists, which led to a decline in its numbers in the natural habitat. Meanwhile, larch trees were exploited for their wood, resin, and tanbark, which further reduced the occurrence of *F. officinalis* and eventually contributed to its status of an endangered species^1^.

Researchers consider *F. officinalis* as one of the medicinal mushroom species that was most commonly used in traditional European medicine^3^. *Fomitopsis officinalis* was thought to be a unique panacea, which was effective in treatment of numerous ailments such as excessive sweating, dizziness, rheumatism, respiratory, digestive, and excretory diseases, cancer^1,2^, hemorrhoids, and dysmenorrhea and as an anti-inflammatory agent. In addition, the mushroom was believed to be a helminthagogue; furthermore, it was believed that large doses of this mushroom would induce vomiting, while smaller ones acted as a diuretic. It also used to be one of the most important anti-inflammatory agents^4^. In the second half of the nineteenth century and in the beginning of the twentieth century, it was mainly used to suppress night sweating in the course of several diseases, particularly in patients with tuberculosis. It was then progressively replaced with agaricin isolated from the mushroom itself. Indeed, modern studies on both crude extracts and compounds isolated from *F. officinalis* demonstrated a wide spectrum of therapeutic effects, including anti-inflammatory, cytotoxic, and antimicrobial activity^3,5^.

To date, studies on *F. officinalis* have focused on the identification of polysaccharides and terpenoids in its fruiting bodies. However, there is still no extensive analysis of other biologically active compounds in both its mycelium and fruiting bodies.

Because of the endangered status of *F. officinalis*, the current study was performed on in vitro cultures of the species grown on a modified liquid medium according to Oddoux. The material was grown in shaken conditions and subsequently in aerial conditions for optimal growth. The taxonomic position of the fungus was confirmed using the PCR method. The study also characterized the biochemical composition of the tested mushroom material with the use of the FTIR method.

To confirm the therapeutic (antioxidant and antiproliferative) activity of *F. officinalis*, the content of indole, phenol, and sterol compounds in lyophilized extracts from mycelium and fruiting bodies was determined using reversed-phase high-performance liquid chromatography (RP-HPLC). Indole, phenol, and sterol compounds were analyzed both quantitatively and qualitatively. Furthermore, trace elements were verified by atomic absorption spectroscopy with flame atomization (F-AAS).

Antioxidant properties of extracts obtained from the species were determined using the DPPH method. The present study also aimed to investigate the antiproliferative activity of mycelium extracts and *F. officinalis* sporocarps against the A549 lung cancer cell line, the DU145 prostate cancer cell line, and the A375 melanoma cell line.

## Results

The aerated conditions of the in vitro cultures contributed to a significant increase in *F. officinalis* biomass growth in the 10-L biofermenter; approximately 12 g dry weight (d.w.) of biomass per liter per day was obtained. This substantially exceeded the amount obtained in in vitro shaken cultures used previously^6^. The resulting biomass from the aerated cultures was used for further genetic, biochemical, and biological research.

### *F. officinalis* mycelium identification

**Figure 1:**
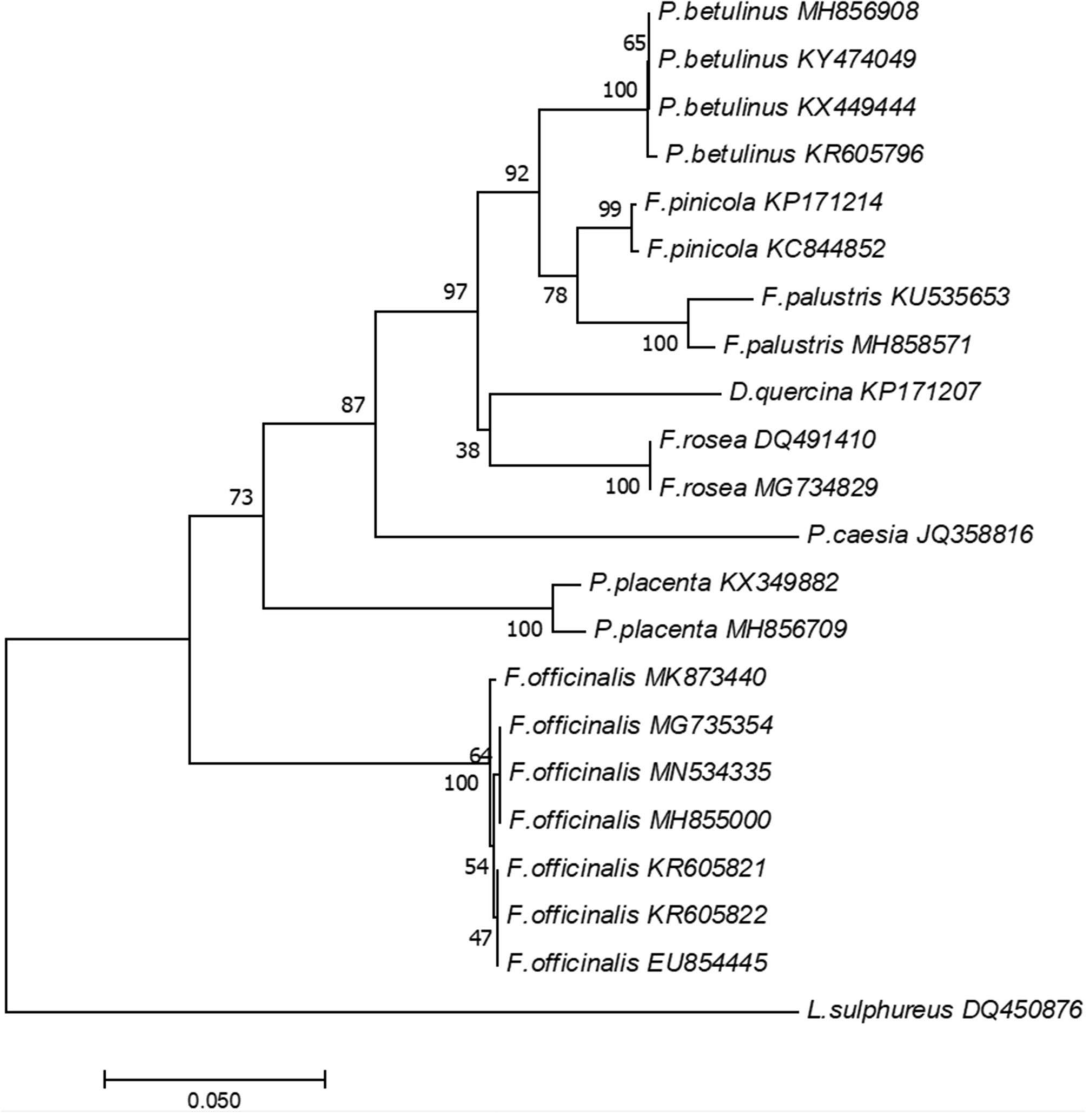
Phylogenetic tree of *F. officinalis* JFo3619 and other closely related strains**.** The evolutionary history was inferred using the N-J method^8^. The analysis involved 22 sequences. The percentages of replicate trees in which the associated taxa clustered together are indicated next to the branches^9^. The evolutionary analyses were computed using the Jukes-Cantor model^10^ and were conducted in MEGA7 software^11^.

A 599-bp PCR fragment of the internal transcribed spacer obtained using ITS1 and ITS4 specific primers was subjected to direct sequencing. Next, it was used to define the phylogenetic position of the tested mushroom. The sequence of this nucleotide fragment revealed over 99% similarity of JFo3619 to *F. officinalis*, as shown in the BLASTN program provided by NCBI. The MN534335 GenBank accession number was granted to the analyzed nucleotide sequence. The N-J algorithm was applied to construct an evolutionary tree for *F. officinalis* JFo3619 and other closely related organisms belonging to the antrodia, i.e. the largest clade of brown rot fungi with ecological and biotechnological importance^7^. The mushroom was grouped together with other *F. officinalis* strains, thus creating a very well-determined cluster with a high bootstrap value (100%) (Fig. 1).

### Biochemical characteristics of fruiting bodies and mycelium extracts. Protein, phenolic compounds, total carbohydrates, and proteolytic activity analyses

The analysis of the protein contents in the fungal extracts showed that the highest amounts of this component in the fruiting bodies of *F. officinalis* (43.43 µg/mL). A higher concentration of phenolic compounds was noted in the fruiting bodies (0.061 mM) than in the mycelium obtained from the in vitro cultures (0.026 mM). A slightly higher level of total carbohydrates was detected in the mycelium biomass (24.8 µg/mL) (Table 1).

**Table 1:** Biochemical parameters of fruiting bodies and mycelium from in vitro cultures of *F. officinalis* extracts.

Sample	Proteins^1^	Phenolic compounds^2^	Total carbohydrates^3^	Antioxidative properties^4^
**Biochemical parameters**				
Fruiting bodies	43.43^a^ ± 2.43	0.061^a^ ± 0.005	23.4^a^ ± 1.85	36.0% ± 5.6
Mycelium	33.86^c^ ± 2.81	0.026^c^ ± 0.005	24.8^a^ ± 3.43	46.0% ± 1.8

^1^The concentration of proteins is expressed in µg/mL.

^2^The content of phenolic compounds is expressed in mM.

^3^The total carbohydrate level is expressed in µg/mL.

^4^The antioxidative potential was expressed as percentage of inhibition of DPPH oxidation. All the results are the average of three replicates of three independent experiments.

Pairs of means with different small letters are significantly different (*p* ≤ 0.05), n = 6; one-way analysis of variance (ANOVA) test was used.

**Figure 2:**
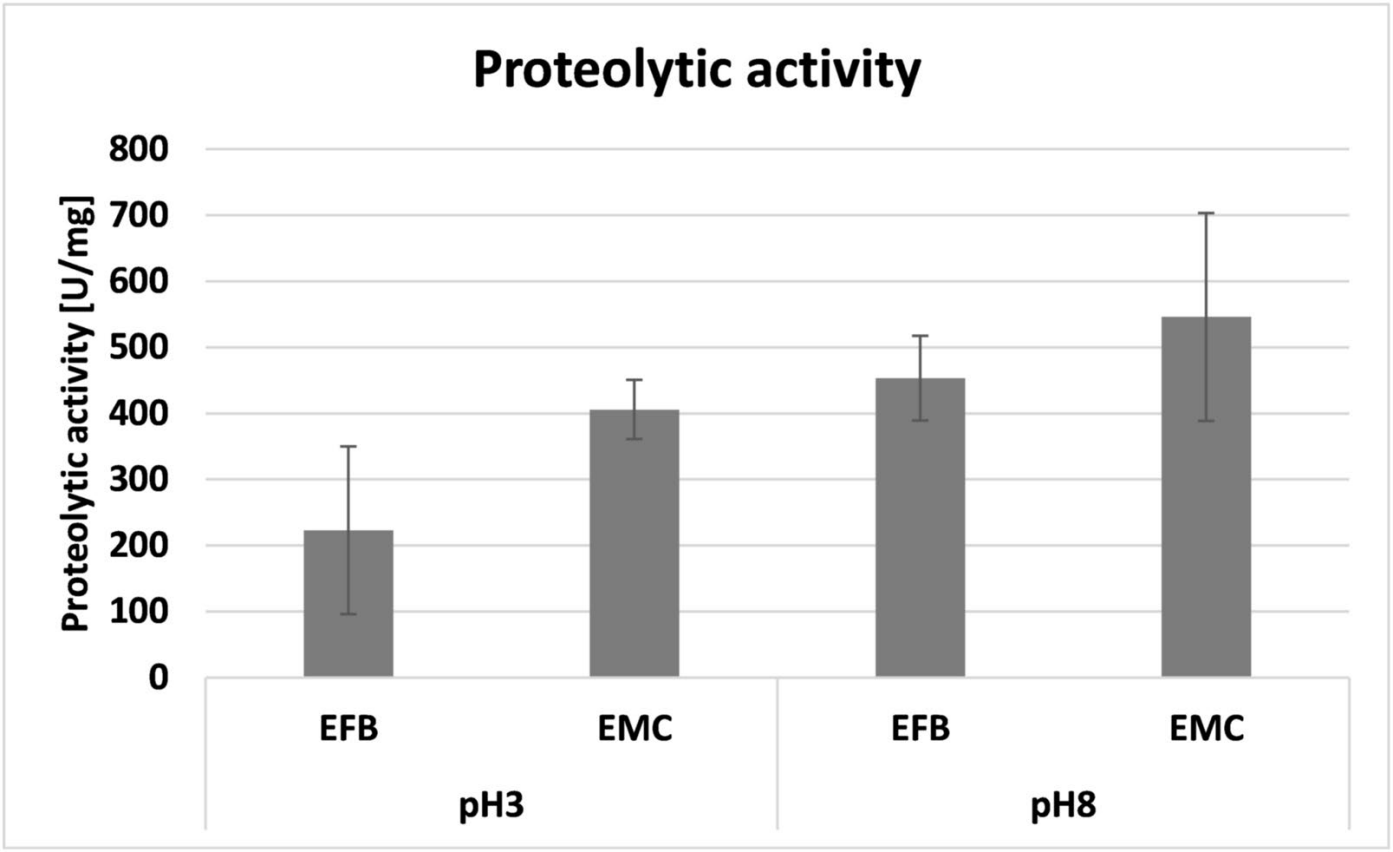
Level of proteolysis determined in acidic (pH 3.5) and alkaline conditions (pH 8.0) using the BSA fluorogenic substrate (EFB: extract of fruiting bodies, EMC: extract of mycelium from an in vitro culture). The specific activity was expressed in relative fluorescence units per minute per microgram of protein (U/min/µg), n = 6.

The proteolytic activity analysis showed effective digestion of the fluorogenic substrate at both pH tested (3.5 and 8.0) (Fig. 2).

The highest proteolytic activity was observed for the extracts of mycelium obtained from the in vitro culture at both pH levels (Fig. 2).

### FTIR analysis

**Figure 3:**
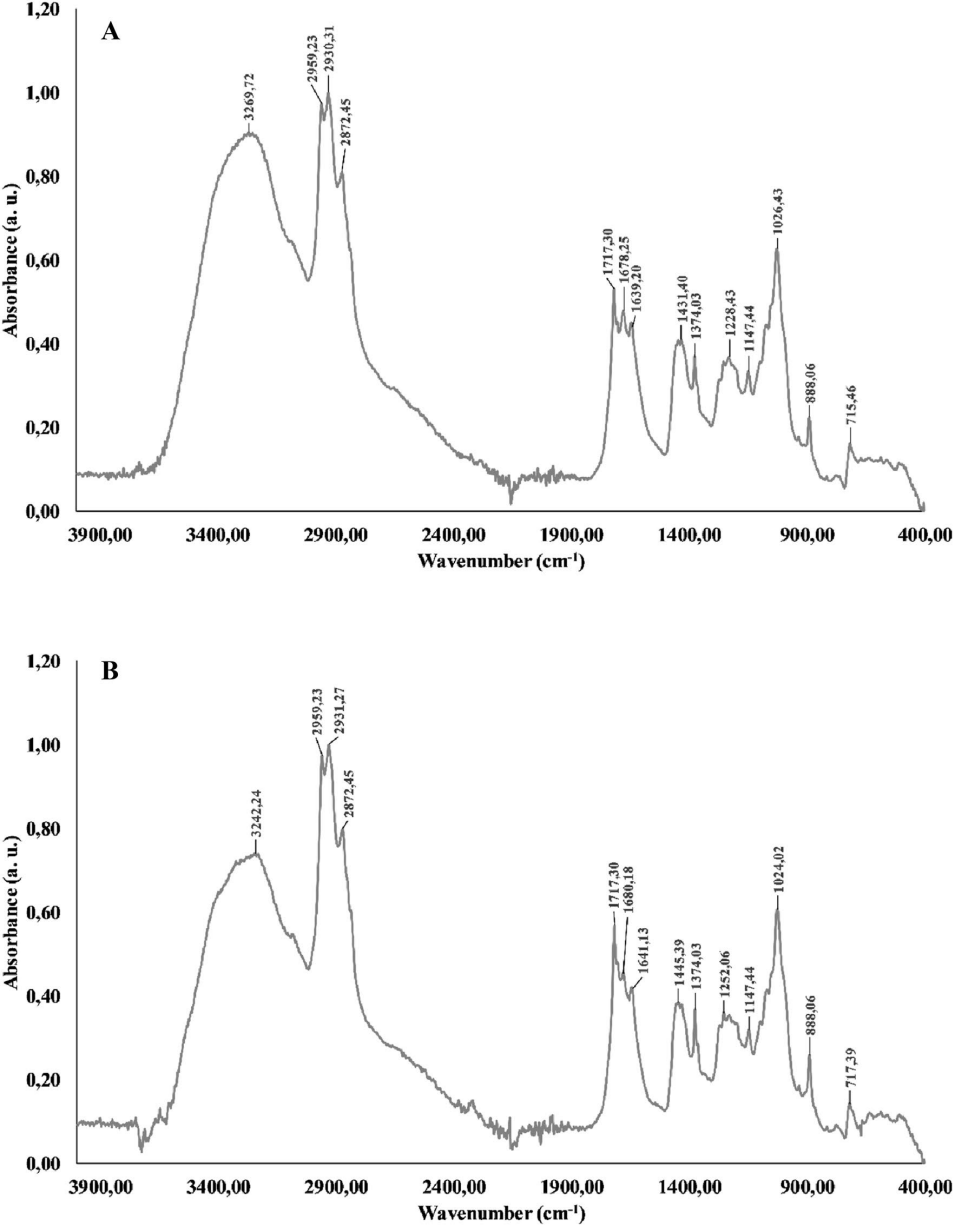
FTIR-ATR spectra of (**A**) extract of fruiting bodies, (**B**) extract of mycelium from in vitro culture).

The spectra from the extracts of fruiting bodies and in vitro mycelial culture shared a high degree of similarity, with three strong bands in the 2960–2850 cm^−1^ range and a medium band in the 1470–1430 cm^−1^ range, suggesting the presence of methylene bridges and/or methyl groups. The absorption at 1150–1020 cm^−1^ may be attributed to various C–O bond vibrations and stretching. Combined with the strong –OH characteristic band peak at 3242 cm^−1^, this finding indicates the presence of polysaccharides, probably glucose derivatives such as maltotriose^12–14^ (Fig. 3).

### Qualitative and quantitative analysis of organic compounds and trace elements

#### Indole compounds

**Figure 4:**
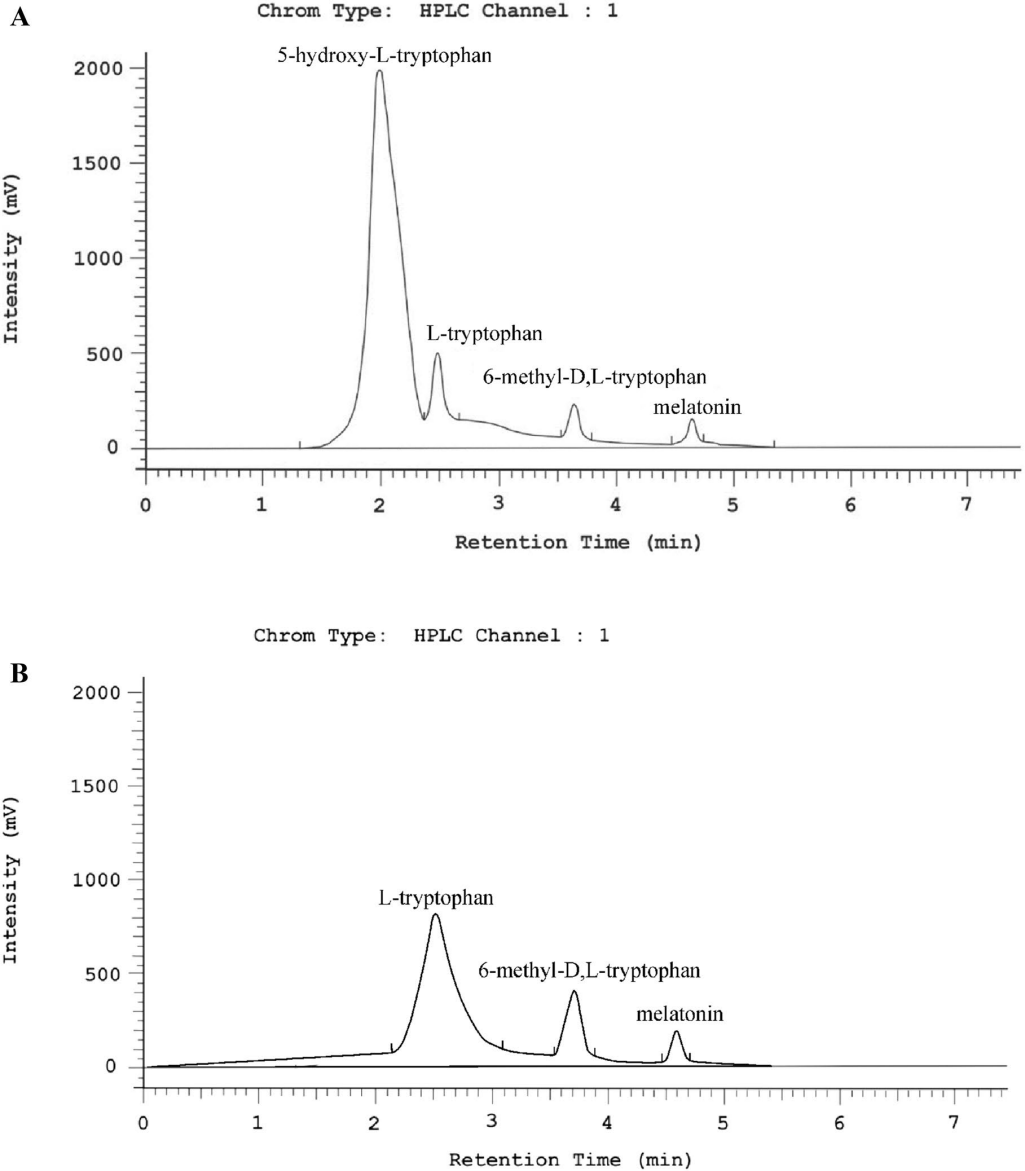
(**A**) Example of a chromatogram of the analyzed indole compounds in *Fomitopsis officinalis* extract from mycelium and (**B**) from fruiting bodies.

The content of four indole compounds (5-hydroxy-l-tryptophan, l-tryptophan, 6-methyl-d,l-tryptophan, and melatonin) was determined qualitatively and quantitatively. An example of a chromatogram of the RP-HPLC separation of indole compounds from *F. officinalis* mycelial cultures and fruiting bodies is shown in Fig. 4.

The highest content of 5-hydroxy-l-tryptophan (517.99 mg/100 g d.w.) was found in the mycelium extract. However, the compound was not found in the fruiting body extract.

l-Tryptophan was found in both types of samples, and its content was 70.08 mg/100 g d.w. in the fruiting body extract and 8.06 mg/100 g d.w. in the mycelium extract. 6-Methyl-d,l-tryptophan and melatonin were also identified in samples from both the fruiting body and mycelium extracts.

The analysis of methanolic extracts confirmed a higher total content of indole compounds in mycelial cultures than in the fruiting bodies of this species. The content of indole compounds determined in the fruiting bodies and mycelia from the in vitro cultures is shown in Table 2.

**Table 2:** Content of selected organic compounds and bioelements in the extracts of fruiting bodies and mycelium of *Fomitopsis officinalis* (mg/100 g d.w.).

Name of substance		Fruiting bodies (mg/100 g d.w.)	Mycelium (mg/100 g d.w.)	
**Indole compounds**				
l-tryptophan		70.08 ± 6.16^a^	8.06 ± 0.96^a^	
6-Methyl-d,l-tryptophan		9.24 ± 1.34^a,b^	0.53 ± 0.06^a,b^	
Melatonin		1.51 ± 0.52^a,b^	0.34 ± 0.03^a,c^	
5-Hydroxy-l-tryptophan		*	517.99 ± 5.29^a,b,c^	
**Phenolic compounds**				
*p*-Hydroxybenzoic acid		0.07 ± 0.00	*	
Catechin		nd	58.37 ± 0.9	
Gallic acid		nd	0.09 ± 0.0	
**Amino acid**				
Phenylalanine		nd	346.48 ± 4.0	
**Sterols**				
Ergosterol		52.97 ± 0.21	102.02 ± 0.44	
Ergosterol peroxide		*	*	
**Bioelements**				
Zn		9.62 ± 0.42^a^	15.34 ± 0.55^a^	
Cu		3.22 ± 0.31^a,b^	0.72 ± 0.04^a,b^	
Fe		4.15 ± 0.12^a,c^	12.06 ± 1.37^a,c^	
Mg		189.5 ± 2.69^a,b,c^	218.9 ± 12.13^a,b,c^	

n = 6; *nd* not detected.

*Below the limit of detection; pairs of means with different small letters are significantly different (p ≤ 0.05); one-way analysis of variance (ANOVA) with a post hoc Tukey test was used.

### Phenolic compounds

**Figure 5:**
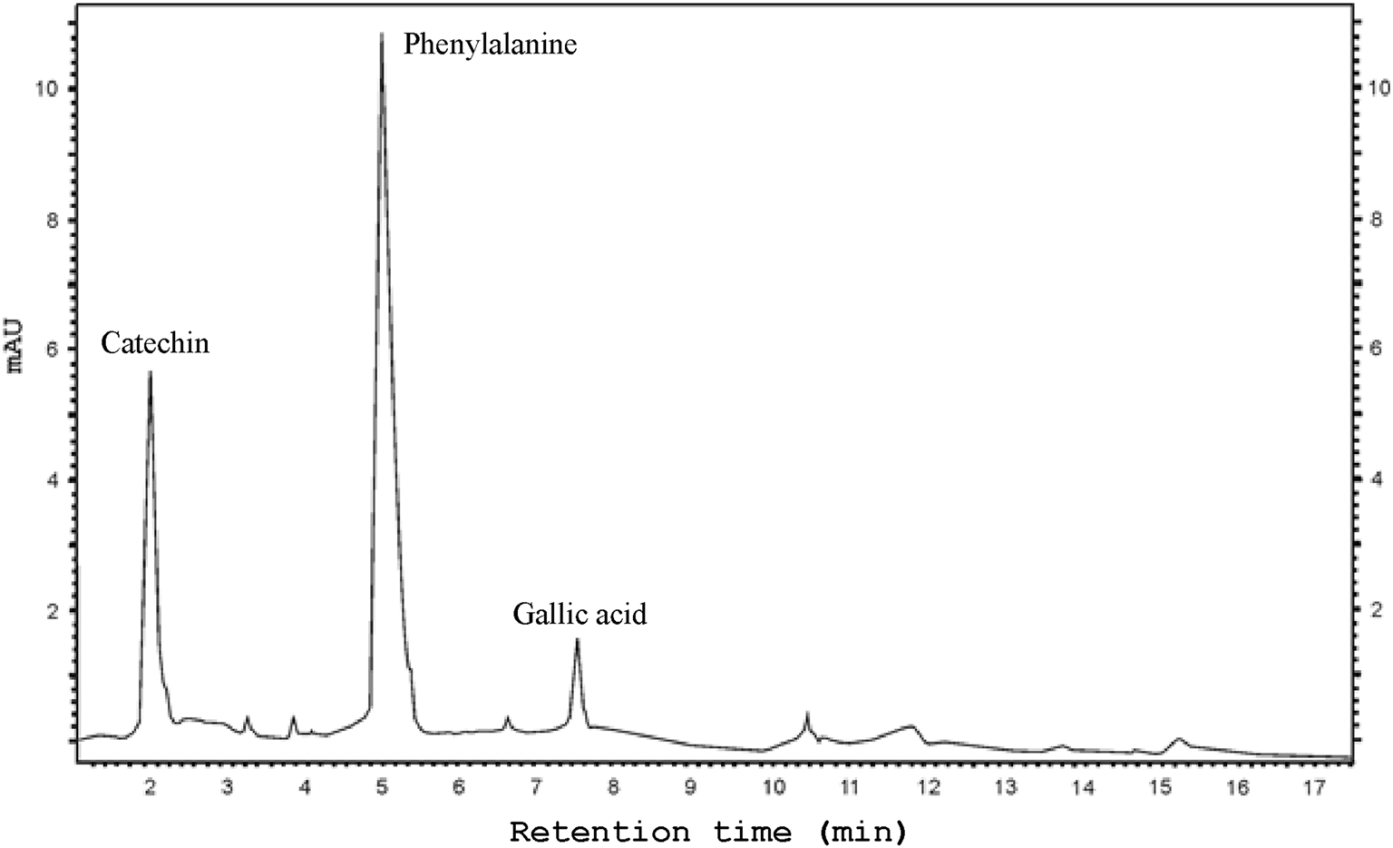
Example of a chromatogram of phenolic compounds detected in the extract of *Fomitopsis officinalis* mycelium.

The presence of two of the 21 phenolic compound standards analyzed in the present study was confirmed (catechin, gallic acid). Additionally, an exogenous amino acid—phenylalanine was identified in the extract from the in vitro* F. officinalis* cultures (Fig. 5).

In the fruiting body extract, however, *p*-hydroxybenzoic acid was detected. Table 2 presents the content of phenolic compounds determined in the methanolic extracts of *F. officinalis* cultures.

### Sterols

Sterol compounds such as ergosterol peroxide (in trace amounts) and ergosterol were identified in the analyzed extracts. Quantitative analysis, whose results are shown in Table 2, demonstrated high content of ergosterol in the mycelium extracts.

### Trace elements

Table 2 shows the content of trace elements analyzed in the extracts of *F. officinalis*. The analysis showed that the mycelium and fruiting bodies of *F. officinalis* accumulated zinc, copper, iron, and magnesium. The average zinc content in the mycelium from the in vitro cultures was 15.34 mg/100 g d.w., which was significantly higher than that in the fruiting bodies (9.62 mg/100 g d.w.).

The iron content was also three times higher in the mycelium (12.06 mg/100 g d.w.) than in the fruiting bodies (4.15 mg 100 g daily). The mycelium obtained from the mycelial cultures had increased content of magnesium ions, compared to that in the fruiting bodies, i.e. 218.9 mg/100 g d.w.

The accumulation of copper only was higher in the fruiting bodies of *F. officinalis* (3.22 mg/100 g d.w.) than in the mycelium from the in vitro cultures (0.72 mg/100 g d.w.).

### Antioxidant activity

The assessment of the antioxidant capacity of the *F. officinalis* methanolic extract with the DPPH radical reduction method revealed that both the investigated mushroom samples could effectively reduce DPPH radicals. The mycelium culture extract showed a higher antioxidant effect (46.4%) than the fruiting body extract (36.0%) (Table 1).

### Antiproliferative activity

The extracts exerted different effects on cancer cell viability (Table 3). The most pronounced effect was shown by extracts obtained from the fruiting bodies.

**Table 3:** IC_50_ represents the ability of the fruiting bodies and mycelium from the in vitro cultures of *Fomitopsis officinalis* extracts to decrease the viability of DU 145, A549, and A375 cell lines after 48 h incubation, measured by the MTT assay.

Extract	IC_50_ (µg/mL)		
	DU 145	A549	A375
Fruiting bodies	11.08	> 100	12.51
Mycelium	10.04	22.58	50.55

The extract obtained from the fruiting bodies exhibited significant activity against two cancer cell lines (DU145 and A375), while no activity was found against the A549 cell line. In contrast, the extract of the *F. officinalis* mycelium from the in vitro cultures not only maintained the anticancer activity of the polypore mushrooms but also exhibited activity against A549.

## Discussion

The dynamics of *F. officinalis* mycelium growth differed from that reported in earlier studies, and the amount of the biomass obtained was higher than that noted previously^6^.

Previously analyzed extracts from the fruiting bodies of the following fungal species: *Agaricus bisporus*, *Armillaria mellea*, *Boletus edulis*, *Boletus badius*, *Cantharellus cibarius*, *Lactarius deliciosus*, *Lactarius deterrimus*, *Leccinum rufum*, *Pleurotus ostreatus*, *Suillus luteus*, and *Tricholoma equestre* were found to contain varying amounts of indole compounds ranging from 0.01 to 34.11 mg/100 g d.w.^15^. The amount of 5-hydroxy-L-tryptophan in the extracts from the *F. officinalis* mycelium was, however, similar to that in the extracts of *A. bisporus* species, which were experimentally digested in an artificial digestive system (from 86.62 to 531 mg 100 g d.w.)^6^.

The total content of indole compounds in the mycelium extracts from the in vitro cultures was 526.39 mg/100 g d.w., while that in the extracts from the fruiting bodies was approximately 80.83 mg/100 g d.w.; this finding indicates that *F. officinalis* may be an important source of therapeutically and physiologically important indole compounds^16^.

With their antioxidant properties and ability to protect cellular structures against oxidative damage, phenolic compounds are known for their biological activities^17^. Gallic acid found in some species of higher fungi has been proven to have anti-inflammatory, antibacterial, antifungal, antiviral, and antioxidant effects^18–22^. The authors systematically observed a correlation between the content of phenolic compounds and the antioxidant activity in mushroom species. Phenolic compounds are regarded as one of the most common antioxidants contained in food. Several scientific studies have confirmed the presence of phenolic acids and their derivatives in representatives of *Basidiomycota*. Phenolic acids such as gallic, *p*-hydroxybenzoic, ferulic, protocatechic, *p*-coumaric, vanillic, and sinapic acids were found, among others, in many fungal species, e.g. *A. mellea*, *B. badius*, *B. edulis*, *C. cibarius*, *L. deliciosus*, and *P. ostreatus*^6,23^. *p*-Hydroxybenzoic, protocatechuic, and vanillic acid were detected in *Fomitopsis pinicola* fruiting bodies^24^. Comparative analysis of *Fomitopsis betulina* fruiting bodies and mycelium showed the presence of syringic, gallic, 5-hydroxybenzoic, and 3,4-dihydrophenylacetic acids^17^. In the present study, the content of phenolic compounds in the *F. officinalis* mycelium from the in vitro cultures was analyzed for the first time. The mycelium extracts showed a significant content of an exogenous amino acid—phenylalanine (34.6 mg/kg d.w.).

In representatives of *Basidomycota*, sterols are a common group of compounds, and ergosterol and ergosterol peroxide are the most representative among them^25^. The results of a research study conducted at the University of Helsinki confirmed the content of these sterols in the range of 625–774 mg/100 g d.w.^26^.

Mushrooms are one of the few sources of ergosterol, which is the basic component of their cell membranes. It practically does not occur in the plant and animal kingdom. It is observed only in a few species of fish, and dairy products usually have to be enriched with vitamin D. Therefore, mushrooms are the main source of the compound. There are two active forms of vitamin D: vitamin D_2_—ergocalciferol and D_3_—cholecalciferol. Under the influence of UV radiation with a wavelength λ = 280–320, ergosterol is transformed into provitamin D_2_, which converts to vitamin D_2_ and ergosterol isomers: lumisterol and tachysterol. Subsequently, vitamin D_2_ is metabolized to a biologically active form—1α, 25-dihydroxyvitamin D_2_. The content of vitamin D in mushroom fruiting bodies depends on conditions prevailing in cultivation sites. Vitamin D increases the absorption of calcium, a process that is particularly important during old age and childhood, because its deficiency may result in osteoporosis and rickets in children. In addition, recent studies have indicated that vitamin D reduces the risk of autoimmune and cardiovascular diseases and malignancies^25^.

Several studies on the chemical composition of *Basidiomycota* representatives have shown high content of ergosterol in mushroom fruiting bodies. Many species of edible mushrooms are particularly widely studied in terms of this characteristic, including *Cantharellus cibarius*, in which the vitamin D_2_ content of the dried fruiting body may reach 1.43 μg/g d.w after several years^27^. *Agaricus bisporus* is another commonly studied species, as it is rich in this vitamin, with the average ergosterol content of up to 61.5 mg/100 g d.w. A high level of ergosterol has also been noted in the fruiting bodies of *Boletus edulis* and is estimated to be 500 mg/100 g d.w., while the vitamin D_2_ content is 200 mg/100 g d.w. The Oriental black mushroom *L. edodes* (Shitake), which is used in the treatment of lifestyle diseases, contains approximately 85 mg ergosterol/100 g d.w.^28^. The ergosterol content measured in the mycelial cultures of *F. officinalis* (102.02 mg/100 g d.w.) was nearly twice that of its fruiting bodies (52.97 mg 100 g d.w.). We obtained similar results in our previous studies, where we compared the content of ergosterol in the fruiting bodies and mycelium in *Fomitopsis betulina*, i.e. 103.99 and 41.29 mg/100 g DM. respectively^17^. In turn, the ergosterol content in another species of the genus *Fomitopsis*, *Fomitopsis pinicola*, was 10,5 mg/100 g d.w. in fruiting bodies^29^. Similar to ergosterol, ergosterol peroxide is a common sterol group compound in the *Basidiomycota* cluster^25^. The steroid is characterized by a wide spectrum of biological activity^27^. It exhibits anti-inflammatory, antimicrobial, antioxidant, cytotoxic, and anticancer effects. The content of ergosterol peroxide depends, among others, on the concentration of reactive oxygen forms and the ratio between its synthesis and re-conversion to ergosterol. It is believed that the presence of antioxidant substances in biomass may affect the ratio of ergosterol to its peroxide form^25^. The content of ergosterol peroxide in the fruiting bodies of *B. edulis* was 30 mg/100 g d.w.^27^. In the *F. officinalis* mycelial cultures, ergosterol peroxide was qualitatively determined in an amount below the limit of detection.

Mushrooms can accumulate larger amounts of trace elements in their fruiting bodies than herbaceous plants^30^. The present results have demonstrated that in vitro culture of *F. officinalis* in aerated conditions yields mycelium with higher content of zinc, magnesium, and iron than fruiting bodies obtained from the natural environment.

Mushrooms from the *Basidiomycota* cluster are a relevant source of compounds with antioxidant properties, such as polyphenols, especially from the flavonoid class. In addition to their ability to capture free radicals and peroxide anions, they protect enzymes that offer antioxidant protection for the body^16,17,23^. They prevent oxidation of LDL fractions and atherosclerosis, simultaneously increasing the level of HDL cholesterol^16,17^. Flavonoids have antimutagenic properties. Phenolic acids are another group of compounds with antioxidant activity^16,17^. Since environmental factors stimulate the synthesis of free radicals, research is being conducted to identify new sources of antioxidants. Researchers are increasingly reporting the possibility of using macrofungi as a source of antioxidants^31^.

Due to the presence of flavonoids and phenolic compounds with proven antioxidant activity in fruiting bodies, *F. officinalis* is also considered a valuable source of compounds with antioxidant properties. Studies on the antioxidant capacity of methanolic extracts of *F. officinalis* confirmed this activity^32,33^. The results of the current study provide additional evidence of the antioxidant activity of this species.

Florczak et al.^31^ conducted a study to assess the content of polyphenols and their antioxidant activity using the DPPH reagent in five species of multifocal fungi: *P. ostreatus*, *Hirneola auricula*, *Flammulina velutipes*, *Polyporus squamosus*, and *Laetiporus sulphureus*. The species were characterized by different contents of polyphenolic compounds, which also depended on the type of extract: aqueous or ethanol. The strongest antioxidant capacity was found in both water and ethanol extracts of *Polyporus squamosus*, while the weakest capacity was noted for the ethanol extract of *Hirneola auricula*. The percentage inhibition of 4 mg/0.1 cm^3^ ethanol extract of *P. squamosus* was 25.36%, 8 mg/0.1 cm^3^ extract yielded 61.70% inhibition, and 12 mg/0.1 cm^3^ extract showed the highest inhibition of 92.55%. It is interesting to note that the content of polyphenolic compounds was not directly correlated with the antioxidant capacity of individual species^31^. Research has also confirmed the significant antioxidant activity of other species of the genus *Fomitopsis*: *Fomitopsis pinicola* and *Fomitopsis betulina*. The antioxidant activity determined with the FRAP method was 55.79 and 24.08 mmol trolox/kg d.w., respectively^24^. Given the results, it can be concluded that the methanol extracts obtained from *F. officinalis* showed antioxidant activity and can be a valuable source of antioxidant compounds.

In this work, the extracts were investigated for antiproliferative activity against three commonly studied cell lines^34–36^. Previous studies showed that the cytotoxic properties of *F. officinalis* were mainly associated with the presence of lanostane saponins^37,38^. The content of these compounds in mycelium and fruiting bodies may be responsible for the differences in the activity of extracts. These considerations require further studies, which would define the role of phytochemicals in the anticancer activity of in vitro cultured mushrooms^37,38^.

In the current study, the content of bioactive substances and the antioxidant and antiproliferative properties of the arboreal medicinal mushroom *F. officinalis* were analyzed using the material obtained from in vitro cultures of fruiting bodies cultivated in shaken and aerated conditions.

The results indicate that *F. officinalis* may be a rich source of indole compounds that are important in both therapeutic and physiological processes. To the best of our knowledge, the presence of 5-hydroxy-l-tryptophan, l-tryptophan, 6-methyl-d,l-tryptophan, and melatonin has been determined both quantitatively and qualitatively for the first time in this species. A surprising and important finding is the particularly high content of 5-hydroxy-l-tryptophan in the mycelium from the in vitro cultures of *F. officinalis*. Furthermore, the phenolic, indole, and sterol compounds as well as trace elements found in the extracts of the *F. officinalis* mycelium and fruiting bodies may contribute to the antioxidant and antiproliferative effects.

The results of this research study, especially the FTIR analyses, showed several similarities in the composition of both extracts; this implies that not only fruiting bodies but also mycelia obtained from in vitro cultures can synthesize metabolites with potential medicinal, pharmaceutical, and cosmetological use. Further experiments should involve testing the antitumor effects of *F. officinalis* extracts in animal models.

## Methods

### Mushroom material

Fruiting bodies were collected from the natural environment in Świętokrzyskie Province (Poland) with the consent of the General Director for Environmental Protection in Warsaw. The mushrooms were identified by Jacek Piętka (Department of Forest Protection, Institute of Forest Sciences, Warsaw University of Life Sciences—SGGW).

Mycelial cultures were obtained from fragments of fruiting bodies placed on Petri dishes with solid agar growth medium. After 4 weeks, the in vitro cultures were passaged to 250 mL of liquid medium according to Oddoux. The laboratory conditions were identical to those in the natural environment in vitro: a 16:8-h light–dark cycle (lighting: 900 lx), temperature: 25 ± 2 °C. The material immersed in liquid medium was shaken at 140 rpm for 4 weeks using an rotary shaker (Altel, Poland).

Subsequently, the mycelium from the in vitro cultures was transferred from Erlenmeyer flasks to an in-house designed 10-L biofermentor and was kept in aerated conditions (sterile air and discharged CO_2_) for further optimal growth. After 10 days, the biomass was separated from the substrate and rinsed four times with distilled water. The biomass was then frozen and lyophilized.

### PCR amplification and sequencing of the mushroom ITS region

Fourteen-day-old mycelia were transferred into a sterile 2.0 mL Lysing Matrix A tube (MP Biomedicals, USA) and homogenized in a FastPrep-24 Homogenizer (MP Biomedicals, USA) in 0.5 mL of spermidine-SDS buffer for 3 × 20 s at 5 m/s rotor speed. After centrifugation (10,000×*g*, 4 °C, 10 min), the supernatant was used for further DNA extraction according to the method of Borges et al.^39^. The quantity and purity of the DNA were evaluated using an ND-1000 spectrophotometer (Thermo Scientific, USA). To confirm the genetic identity of the fungus, the ribosomal RNA gene fragment was amplified using the universal primers ITS1 (TCCGTAGGTGAACCTGCGG) and ITS4 (TCCTCCGCTTATTGATATGC) as described by White et al.^40^. PCR was performed in a T-Personal thermal cycler (Biometra, Germany) using DreamTaq Green PCR Master Mix (Thermo Scientific, USA). The amplified region was analyzed by direct sequencing of the PCR product. Automatic sequencing was performed using the BigDye Terminator Cycle Sequencing Kit and ABI PRISM 310/3730 XL sequencers (Applied Biosystems, USA). Data from ITS sequencing were analyzed with ChromasPro v.1.6 software (Technelysium Pty Ltd, Australia). Database search was performed with the BLAST program at the National Center for Biotechnology Information (Bethesda, MD, USA)^41^. Multiple DNA sequence alignments were performed with the Clustal-W algorithm^42^. The neighbor-joining (NJ) algorithm was used to construct a phylogenetic tree as implemented in MEGA v.7.0 software^11^. The topology of the tree was evaluated by bootstrap analysis of the sequence data based on 1000 random resampling.

### Biochemical analysis

#### Sample preparation

Biochemical analyses were carried out on two types of mushroom extracts: methanol extract of *F. officinalis* fruiting bodies obtained from the natural environment and methanol extract of biomass produced in in vitro conditions. Lyophilized preparations were dissolved at the ratio of 1 mg of lyophilizate/1 mL of methanol. The samples of the biological material were used in all the biochemical analyses.

#### Determination of the concentration of protein and phenolic compounds (PC), total carbohydrate (TC) level, and proteolytic activity

The protein concentration in the samples was determined using Coomassie brilliant blue (G-250) dye with bovine serum albumin as a standard. The concentration of proteins was expressed in µg/mL^43^. The PC concentration was determined with the DASA test using diazosulfanilamide (SA) as a reaction substrate^44^. The absorbances obtained were recorded at 500 nm at room temperature and compared with the data from the calibration curve (y = 6.85x − 0.0218, R^2^ = 0.999). The TC content in the samples was determined with the phenol–sulfuric acid-based method. D-glucose served as a standard substrate for the calibration curve, and the results were expressed in µg/mL^45^. The proteolytic activity of the samples was estimated using the fluorescent micro-method with BSA-BIODYPY heavily labeled bovine serum albumin (Invitrogen DQ Red BSA, Thermo Scientific, USA) as a reaction substrate. The analysis was conducted at two different pH values (3.5 and 8.0). The reaction mixture contained 50 µL (50 µg/mL) of the reaction substrate, 50 µL (1 mg/mL) of the sample, and 100 µL of McIlvaine buffer (pH 3.5 or 8.0). The kinetic substrate digestion was determined for 30 min (excitation 590 nm and emission 620 nm) using a Tecan Infinite m200 plate reader. The specific activity of proteases was calculated based on the fluorescence increase corresponding to the substrate digestion rate and expressed in relative fluorescence units (U) per minute per µg of protein.

#### FTIR analysis

Fourier-transform infrared spectroscopy of the methanol extracts of the fruiting bodies and biomass of *F. officinalis* produced in in vitro conditions was performed using a Nicolet 8700 A spectrometer (Thermo Scientific, USA) equipped with an FT Raman Nicolet NXR module in the 4000–400 cm^−1^ wavelength range.

#### Determination of indole and phenolic compound

The lyophilized biomass (5 g) was extracted five times with 100 mL of methanol using ultrasound at a frequency of 49 kHz for 30 min (Sonic-2, Polsonic, Poland). Qualitative and quantitative HPLC–DAD analyses were performed according to previously published protocols: indole compounds^6,17^ and phenolic compounds^17^.

#### Determination of sterols

The lyophilized biomass (5 g) was extracted with a mixture of methanol/dichloromethane 75:25 (v/v). The mixture was sonicated at 40 kHz for 10 min. After 2 h, the extract was centrifuged at 12,000 rpm for 5 min and decanted. Qualitative and quantitative HPLC–DAD analyses were performed according to the previously published protocols^17^.

#### Determination of trace elements

The lyophilized mushroom materials were weighed in amounts of 0.2 g each with an accuracy of 0.01 g. After weighing, the material was transferred to Teflon vessels, to which 5 mL of concentrated 65% HNO_3_ (Merck, Germany) and 2 mL of 30% H_2_O_2_ (Merck, Germany) were added. Mineralization was conducted in the Magnum II microwave apparatus (Ertec, Poland) in three stages (10 min each), with 100% power. The temperature of the device was maintained at 295 °C. After the mineralization process, the solutions were transferred to quartz evaporators using water distilled four times. The residue of the reagents and water was evaporated on a heating plate at 150 °C. The residue of the mineralized sample obtained after evaporation was quantitatively transferred to 10-mL volumetric flasks with water distilled four times. The samples prepared in this way were analyzed with the use of Flame Atomic Absorption Spectrometry (F-AAS). Mg, Zn, Cu, and Fe were analyzed using an iCE3300 Thermo Scientific spectrophotometer (USA). Standard curves were prepared for each of the tested bioelements using various concentrations of the standards (Merck, Germany). The mineralization process and F-AAS analysis were conducted following the previously published methods^46^.

#### Antioxidant activity

The DPPH method performed according to the procedure proposed by Paduch et al.^47^ was used to determine the antioxidative properties of all mushroom samples. The procedure was also described in detail by Jaszek et al.^48^ and Matuszewska et al.^49^.

### Antiproliferative activity determination

#### Cell cultures

The A549 (ATCC) lung cancer cell line, the DU145 (ATCC) prostate cancer cell line, and the A375 (ATCC) melanoma cell line were used. Cells were grown in culture flasks in standard conditions of temperature (37 °C) and CO_2_ concentration (5%). The A549 and A375 cell lines were cultured in DMEM medium (Gibco) supplemented with 10% fetal bovine serum (FBS) (Gibco, Ireland) and antibiotics (Lonza, Switzerland), while the DU145 cells were cultured in DMEM-F12 medium (Gibco, Ireland) supplemented with 10% FBS and antibiotics. Doxorubicin hydrochloride (> 95% purity) was purchased from Enzo Life Sciences, USA.

#### Cell viability

Cells were seeded on 96-multiwell plates at the density of 5000 cells/well. After 24 h, the cells were incubated with extracts dissolved in DMSO to a final concentration of 0–100 µg/mL, and the plates were gently mixed. Negative (vehicle) and positive (doxorubicin hydrochloride) controls were included. After 48 h incubation, the MTT solution (5 mg/mL) was added to the medium. After 3 h, when black crystals of formazan appeared at the bottom of the wells, the medium was removed and formazan was dissolved in DMSO. Absorbance was read on a multiwell reader (Spectra Max iD3, Molecular Devices, USA) at 570 nm. Cell viability was determined by dividing the absorbance of the experimental wells by the absorbance of the control wells (× 100%). Three separate repeats of the experiment were performed. DMSO had no significant effect on cell viability. IC_50_ was calculated using GraphPad Prism 7.01^50^.

#### Statistical analysis

One-way analysis of variance (ANOVA) with a post hoc Tukey test was used to validate the statistical hypotheses at *p* = 0.05 for all tests. All statistical analyses were performed using the Statistica software.
